# Supplementary material for: BINSEQ: A family of high-performance binary formats for nucleotide sequences
Source: PLoS Comput Biol. 2026 May 28;22(5):e1014181. doi: 10.1371/journal.pcbi.1014181 (PMC13232939; doi:10.1371/journal.pcbi.1014181)
Supplement: S1 Table — Standard nucleotides use two-bit encoding. In four-bit mode, these values are preserved in the least significant bits with leading zeros, while N requires the full four-bit representation. (PDF) [file pcbi.1014181.s001.pdf]

S1 Table: Nucleotide Encoding. Standard nucleotides use two-bit encoding. In four-bit mode, these values are preserved in the least significant bits with leading zeros, while N requires the full four-bit representation.

**Nucleotide**	**two-bit**	**four-bit**
A	00	0000
C	01	0001
G	10	0010
T	11	0011
N	—	1111
