# Supplementary material for: BINSEQ: A family of high-performance binary formats for nucleotide sequences
Source: PLoS Comput Biol. 2026 May 28;22(5):e1014181. doi: 10.1371/journal.pcbi.1014181 (PMC13232939; doi:10.1371/journal.pcbi.1014181)
Supplement: S2 Table — Complete specification of the 32-byte BQ header including field offsets, sizes, types, and descriptions for magic number, version, sequence lengths, bit encoding, flags, and reserved bytes. (PDF) [file pcbi.1014181.s002.pdf]

S2 Table: BQ Header Structure (32 bytes)

Offset	Size (bytes)	Field	Type	Description
0	4	magic	uint32	Magic number (0x42534551)
4	1	version	uint8	Format version (currently 2)
5	4	slen	uint32	Sequence length (primary)
9	4	xlen	uint32	Sequence length (secondary)
13	1	bits	uint8	Number of bits per nucleotide (2, 4)
14	1	flags	bool	Records are prefixed by a flag uint64
15	17	reserved	[uint8]	Reserved for future extensions
