# Supplementary material for: BINSEQ: A family of high-performance binary formats for nucleotide sequences
Source: PLoS Comput Biol. 2026 May 28;22(5):e1014181. doi: 10.1371/journal.pcbi.1014181 (PMC13232939; doi:10.1371/journal.pcbi.1014181)
Supplement: S3 Table — Complete specification of the 32-byte VBQ header including format version, virtual block size, and boolean flags indicating presence of quality scores, compression, paired-end reads, sequence headers, and record flags. (PDF) [file pcbi.1014181.s003.pdf]

S3 Table: VBQ Header Structure (32 bytes)

Offset	Size (bytes)	Field	Type	Description
0	4	magic	uint32	Magic number (0x51455356)
4	1	format	uint8	Format version (currently 1)
5	8	block	uint64	Virtual size of all blocks in bytes
13	1	qual	bool	Records include quality scores
14	1	compressed	bool	Blocks are compressed
15	1	paired	bool	Records consist of sequence pairs
16	1	bits	uint8	Number of bits per nucleotide (2, 4)
17	1	headers	bool	Records include sequence headers
18	1	flags	bool	Records are prefixed by a flag uint64
19	13	reserved	[uint8]	Reserved bytes for future extensions
