# Supplementary material for: BINSEQ: A family of high-performance binary formats for nucleotide sequences
Source: PLoS Comput Biol. 2026 May 28;22(5):e1014181. doi: 10.1371/journal.pcbi.1014181 (PMC13232939; doi:10.1371/journal.pcbi.1014181)
Supplement: S4 Table — Specification of the 32-byte block header structure including magic number, true block size (accounting for compression), number of records in block, and reserved bytes for future extensions. (PDF) [file pcbi.1014181.s004.pdf]

S4 Table: VBQ Block Header (32 bytes)

**Offset**	**Size (bytes)**	**Field**	**Type**	**Description**
0	8	magic	uint64	Magic number (0x5145534B434F4C42)
8	8	size	uint64	True size of record block (can vary if compressed)
16	4	records	uint32	Number of records in block
20	12	reserved	[uint8]	Reserved bytes for future extensions
