# Supplementary material for: BINSEQ: A family of high-performance binary formats for nucleotide sequences
Source: PLoS Comput Biol. 2026 May 28;22(5):e1014181. doi: 10.1371/journal.pcbi.1014181 (PMC13232939; doi:10.1371/journal.pcbi.1014181)
Supplement: S5 Table — Detailed field-level specification of variable-length VBQ records including flags, sequence lengths, encoded nucleotide buffers, quality scores, and sequence headers for both primary and extended (paired-end) sequences. (PDF) [file pcbi.1014181.s005.pdf]

S5 Table: VBQ Record. B represents the number of bits per word and is 32 for two-bit encodings or 16 for four-bit.

**Field**	**Type**	**Size (bytes)**	**Description**
flag	uint64	8	Binary flag for the record
slen	uint64	8	Primary sequence length
xlen	uint64	8	Extended sequence length
sbuf	[uint64]	$\lceil \text{slen}/B \rceil$	Encoded primary sequence
squal	[uint8]	slen if paired else 0	Primary sequence quality scores
sheader	[uint8]	8 + Variable	Sequence Header. The first uint64 (8 bytes) defines the length in bytes of the header. The remaining bytes contain the header data.
xbuf	[uint64]	$\lceil \text{xlen}/B \rceil$	Encoded extended sequence
xqual	[uint8]	xlen if paired else 0	Extended sequence quality scores
xheader	[uint8]	8 + Variable	Sequence Header. The first uint64 (8 bytes) defines the length in bytes of the header. The remaining bytes contain the header data.
