# Supplementary material for: BINSEQ: A family of high-performance binary formats for nucleotide sequences
Source: PLoS Comput Biol. 2026 May 28;22(5):e1014181. doi: 10.1371/journal.pcbi.1014181 (PMC13232939; doi:10.1371/journal.pcbi.1014181)
Supplement: S6 Table — Specification of the 32-byte index header structure including magic number, file size verification field, and reserved bytes for future extensions. (PDF) [file pcbi.1014181.s006.pdf]

S6 Table: VBQ Index Header (32 bytes)

**Offset**	**Size (bytes)**	**Field**	**Type**	**Description**
0	8	magic	uint64	Magic number (0x5845444e49514256)
8	8	bytes	uint64	Number of bytes in the file pair)
16	16	reserved	[uint8]	Reserved bytes for future extensions
