# Supplementary material for: BINSEQ: A family of high-performance binary formats for nucleotide sequences
Source: PLoS Comput Biol. 2026 May 28;22(5):e1014181. doi: 10.1371/journal.pcbi.1014181 (PMC13232939; doi:10.1371/journal.pcbi.1014181)
Supplement: S7 Table — Specification of the 32-byte index range structure describing block locations, including file offset, block size, number of records per block, cumulative record count, and reserved bytes. (PDF) [file pcbi.1014181.s007.pdf]

S7 Table: VBQ Index Range (32 bytes)

Offset	Size (bytes)	Field	Type	Description
0	8	offset	uint64	File offset of block start (bytes)
8	8	len	uint64	Size of the block (bytes)
16	4	records	uint64	Number of records in block
20	4	cumulative	uint64	Number of records before block
24	8	reserved	[uint8]	Reserved bytes for future extensions
