# Supplementary material for: BINSEQ: A family of high-performance binary formats for nucleotide sequences
Source: PLoS Comput Biol. 2026 May 28;22(5):e1014181. doi: 10.1371/journal.pcbi.1014181 (PMC13232939; doi:10.1371/journal.pcbi.1014181)
Supplement: S8 Table — Description of the map-reduce interface for parallel processing including the process_record map function for individual record processing and the on_batch_complete reduce function for batch aggregation. (PDF) [file pcbi.1014181.s008.pdf]

S8 Table: Parallel Processing Hooks

Hook	Type	Description
process_record	map	Process a single record (primary / extended)
on_batch_complete	reduce	Called when a batch of records has completed processing
