# Supplementary material for: BINSEQ: A family of high-performance binary formats for nucleotide sequences
Source: PLoS Comput Biol. 2026 May 28;22(5):e1014181. doi: 10.1371/journal.pcbi.1014181 (PMC13232939; doi:10.1371/journal.pcbi.1014181)
Supplement: Attachment — Submitted filename: review_responses.pdf [file pcbi.1014181.s010.pdf]

Review Responses

Introduction and General Changes

We'd like to thank the reviewers for their insightful comments and suggestions. We've responded to each of their points below, but would like to describe some top-level changes to BINSEQ that address and intersect with many of their comments.

Renaming BINSEQ variants

While not directly mentioned by the reviewers, we've had comments that using BINSEQ as the format name and as one of the variants was confusing. We've since renamed the variants BQ and VBQ respectively.

Support for Four-Bit Encoding

We've added support for four-bit encoding for all variants of BINSEQ. Both BQ and VBQ now support four-bit encodings (currently just N but with plans to support all IUPAC in the future).

Support for Optional Sequence Headers in VBQ

We've added support for optional sequence headers in VBQ which allows for complete lossless support of FASTQ when used with four-bit encoding.

Consolidating the VBQ Index File into the VBQ File

We've consolidated the VBQ index file into the VBQ file itself so that there are no extraneous files.

Implementation Changes and Fixes

We've also caught some implementation errors in our original manuscript when rerunning our benchmarking.

Sequence Access

In the sequence access we found that we were actually incorrectly subscribing threads in the parallel implementations of our sequence access benchmark. We've since fixed this and found that we significantly improve our throughput. Figure 2 and Table 2 have been updated to include the new benchmarking (with corrected subscription) and the relevant text in section 3.1 has been updated.

We've also correctly noted that this benchmarking was performed locally on a MacBook while the higher-thread counts comparisons (k-mer, alignment) were performed on an HPC.

K-mer Mapping

In the k-mer mapping algorithm we realized that our implementation was not as described in the methods and was sub-optimal and adding more per-sequence-load. We fixed the implementation and found throughput gains for both BINSEQ and FASTQ implementations, though we were able to show significantly more improvement of thread-scaling as a result. Figure 3 has been updated to address this and relevant text in section 3.2 has been updated

Reviewer Responses

All reviewer text is italicized and their comments are additionally bolded and in red font.

Reviewer 1

Teyssier and Dobin described BINSEQ and VBINSEQ, two binary formats for storing NGS reads with I/O performace being a primary focus. BINSEQ is designed for reads of fixed length while VBINSEQ for reads of variable length and optionally keeps base quality. Neither format stores read names. The format

design is straightforward and the implementation is well engineered. The authors BINSEQ greatly outperforms other formats and associated tools.

Critical comments are as follows:

> ***As a general comment, I see BINSEQ will have limited use cases. Base quality is essential to variant calling. This is why Illumina tried to reduce quality resolution but still decided to use 2 bits per quality. Base quality is unimportant to RNA-seq quantification, but read lengths could be variable due to trimming. BINSEQ wouldn't work in this case, either.***

We appreciate the reviewer raising this point about use cases. We agree that BQ's design choices optimize it for specific applications rather than universal use. However, we note that the applications it does serve, particularly single-cell RNA sequencing and amplicon-like sequencing, represent a substantial and growing fraction of contemporary sequencing data.

For the broader range of applications mentioned by the reviewer (variant calling, RNA-seq post-filtering with variable length reads), VBQ provides the necessary flexibility while maintaining significant performance advantages over compressed FASTX, as demonstrated in our benchmarks. We've added some additional text to the sequence access results which discusses these trade-offs.

> ***As the more flexible format, VBINSEQ should optionally store read names. Illumina read names carry information and are still used for marking optical duplicates if I am right. Probably removing names will have little impact to analysis but if the authors want VBINSEQ to be an archival format, a lossless mode would be essential to data centers. The authors can use the flag field to indicate the presence/absence of read names.***

This is a great addition - users have also asked for read names to store genome contig information. This is now available on vbq files (tracked in the header) and can store both read names in the data. We have also added an `--archive` mode to `bqtools` which stores read names, four-bit nucleotides, and quality scores for records in a lossless way. We've additionally added some text to the sequence access results which discusses these trade-offs.

> ***How are non-A/C/G/T bases stored? The UCSC 2-bit format uses a sparse vector to keep positions of non-A/C/G/T bases. The authors can do the same for sequences with "N" and use a flag bit to indicate the presence/absence of this information.***

The reviewer raises an excellent point about handling of non-ACGT bases. In our current implementation, the two-bit encoding does not preserve this information. We considered implementing a sparse vector approach similar to the UCSC 2-bit format but determined that for billion-record files, the overhead of tracking these positions would significantly impact both file size and access performance. This would undermine the core design goals of BINSEQ.

To address this limitation, however, we have implemented two complementary solutions:

1. Support for four-bit encoding when preservation of ambiguous bases is required
2. Configurable policies for handling invalid nucleotides (in bqtools via the –policy flag) that allow users to clearly specify how these nucleotides should be treated (random substitution, fixed replacement, sequence skipping, or error reporting).

We've updated the text to reflect four-bit support and updated the nucleotide encoding table in the supplemental as well.

> ***Zstd and pigz (a popular gzip alternative) support multi-threading. Please evaluate their multi-threading mode in Table 2. By the way, on the Time column, better to use 0.21, 0.05, 0.86, etc for timing. This is easier to read in my opinion.***

We thank the reviewer for this suggestion, but should clarify that while while zstd and pigz do support multi-threading, this capability applies only to compression/encoding. Our benchmarks measure decompression/decoding throughput, which remains single-threaded in both implementations.

We have also replaced the timing statistics with non-scientific notation for clarity as suggested.

> ***What data is used for benchmarking? Please specify the resolution of base quality, the format of read names, the average read depth and whether input reads are sorted by coordinate. Some of these factors may greatly affect compression ratio.***

We appreciate the reviewer's request for additional experimental details and acknowledge that we had not explained it well in the original manuscript. We have added comprehensive information about the benchmark data to Section 2.3.1. Briefly: base quality was uniformly set to '?' to avoid making assumptions about quality score distributions while providing favorable conditions for existing compression algorithms. Nucleotide sequences were generated with uniform random distribution (25% each of A, C, G, T) and were unsorted—representing a challenging case for compression algorithms. Read names followed the simple format @seq.{idx} where idx indicates record position. These choices were made to create reproducible benchmarks that do not favor BINSEQ through unrealistic data characteristics. We note that real sequencing data often exhibits structure that would further improve compression ratios for all formats.

We note that real sequencing data often exhibits non-random structure (e.g., GC content bias, quality score distributions) that would improve compression ratios for all formats, making our benchmark a conservative estimate of relative performance.

Reviewer 2

In this manuscript, the authors present a family of two binary file formats for nucleotide sequences. First, the BINSEQ format, which is designed for storing sequences of a fixed length. Second, the VBINSEQ format, an extension to BINSEQ that can contain sequences of variable length. With the development of these formats, the authors seek to increase the speed at which sequences can be accessed, and hence the speed of computations reading that data. To showcase this improvement, the authors compare the speed and storage footprint of their formats to other standard formats, and particularly the FASTQ/FASTA formats, which currently serve as the de facto default formats for the storage, sharing, and archiving of sequence data.

Overall, I feel that the authors created suitable and fair tests to assess the efficacy of the BINSEQ formats. Furthermore their tests showcase that these formats achieve their goal of increasing the parallelisation efficiency of operations, while maintaining a storage footprint similar in size to FASTQ files. While this is not the first attempt to improve on sequence data formats, it is the first approach I have seen to focus on improving the access speed of the data, instead of only their storage footprint. With the ever-increasing volume of sequencing data being produced, storing this data in optimised data formats that are interoperable and easily convertible is more relevant than ever. However, there are design decisions made in the creation of these formats that might severely limit their applicability across

the genomics field. Namely, the current omission of quality scores, sequence identifiers, and uncertain base calls and/or Ns in the stored sequences. Admittedly, my own experience is limited to low-coverage ILLUMINA data from human DNA, so I acknowledge that perhaps the omitted information is less important in when working with other types of sequencing data. Should changes to this format allow for the inclusion of the omitted information (thus making it fully equivalent to FASTQ), BINSEQ would become a valuable data format for genomics in the age of HPCs and cloud computing.

Major comments:

> ***It is not entirely clear what niche the BINSEQ format currently fills. The lack of any quality scores makes it suboptimal for storing raw reads (like a FASTQ does), while the specification that sequences must be of equal length makes it unsuitable for storing reference sequences composing multiple contigs (like a FASTA does).***

We thank the reviewer for raising this important concern about the scope of applicability. The reviewer is correct that BQ is optimized for specific use cases rather than universal application. It is particularly well-suited for fixed-length read scenarios where quality scores are not required for downstream analysis, including single-cell RNA sequencing and amplicon sequencing, both representing substantial and growing segments of sequencing data production.

We have addressed the broader concerns in two ways: First, VBQ extends the format to variable-length sequences with optional quality scores, making it suitable for a wider range of applications. Second, based on reviewer feedback, we have implemented an –archive mode in bqtools that preserves read names, uses four-bit nucleotide encoding, and retains quality scores, providing a lossless storage option.

We have expanded the Results and Discussion to more clearly articulate that there are trade-offs at an application-specific level for including or excluding specific information in the format.

> ***Considering that this file format is designed for storing reads before any alignment, the decision to not allow for Ns in the nucleotide sequences in either format is dangerous when working with lower-coverage data. This becomes a bigger issue in the BINSEQ format, where quality scores are not accounted for.***

This is a valid concern about the handling of ambiguous bases, particularly for low-coverage sequencing applications where N bases may carry important quality information.

We have addressed this limitation through the four-bit encoding option now available in both BQ and VBQ. When four-bit encoding is enabled, Ns are preserved exactly as in the input. This provides users with a clear choice based on their application requirements: two-bit encoding maximizes speed and minimizes storage for applications where ambiguous bases are rare or unimportant, while four-bit encoding preserves all base information at the cost of modest increases in storage and decoding time.

For VBQ specifically, four-bit encoding combined with quality score retention and header retention provides complete lossless preservation of sequencing reads, making it suitable even for low-coverage variant calling applications where the reviewer's concerns are most acute.

> ***More broadly, I am very skeptical of the decision to drop quality scores from the BINSEQ format specification altogether. The authors argue that "quality scores and sequence identifiers [are] frequently ignored by modern bioinformatics tools", and hence can be***

dropped from BINSEQ files entirely. This might be okay to do if everything goes according to plan during data generation, but it allows for no troublshooting should anything go wrong (e.g. if some part of a flowcell produces poor quality data).

We appreciate the reviewer raising this consideration about quality scores in troubleshooting workflows. We respectfully offer a perspective informed by our experience with production genomics pipelines. In practice, quality control and troubleshooting of sequencing data typically occurs at the instrument or run level rather than during downstream bioinformatics analyses. Modern sequencing platforms provide extensive QC metrics and reports that identify problematic runs, flowcell regions, or lanes before sequencing records reach the analysis pipeline. For well-established protocols, particularly applications like RNA-seq and single-cell sequencing where base quality is generally high, issues requiring read-level quality score examination during analysis are relatively uncommon.

Our design philosophy reflects this observation: BQ is optimized for production-scale analyses where sequence data quality has been validated and quality scores provide limited additional value for the specific downstream application (e.g., UMI counting in single-cell RNA-seq, k-mer-based methods, transcript quantification). For applications where quality scores remain important, such as variant calling from low-coverage data or development of novel protocols, VBQ provides full quality score support with optional compression.

Users can retain FASTQ or quality-score-enabled VBQ during initial validation, then convert to more optimized formats for production analyses once data quality is established. This approach balances data completeness during development with processing efficiency during production. Ultimately, the decision of what data to include or exclude should be left up to practitioners and the choice to drop quality scores or ambiguous bases for significant throughput gains is truly application dependent.

We have expanded our Discussion section to reflect these trade-offs and note on application-specific usage of specific BINSEQ variants.

Minor comments:

4. ***Please ensure the legends of tables and figures are accurately describing the content. For example: a. Table2: The legend states the times given are in milliseconds, but the unit stated is (s). b. Figure 4e: The legend states this plot shows the speedup of VBINSEQ, but the figure includes results for short-read spliced alignment, which was only ran with BINSEQ, and not VBINSEQ.***

We thank the reviewer for catching these errors. Both have been corrected:

1. Table 2 legend now correctly states times are in seconds
2. Figure 4e legend has been corrected to reflect that short-read spliced alignment uses BQ.

Reviewer 3

In this manuscript, Teyssier and Dobin introduce the BINSEQ format (both the simple binseq and more flexible vbinseq) formats. They describe the format in detail, argue for its utility compared to the FASTQ format for storing primary sequencing data; especially in the context of high-throughput analysis tasks such as k-mer counting and read alignment via case studies with modified versions of existing tools. They also introduce a suite of tools for handling and converting to and from these formats. Overall, I share the concerns of the authors about the fundamental way in which the FASTQ format is ill-suited for modern parallel processing tasks, and shudder to think how much time (both CPU time and wall-clock time) has been wasted simply parsing a format that was never well-designed for the purposes of

parsing by a computer. The BINSEQ format is a big step up from FASTQ but ultimately, as the authors themselves suggest, whether or not a true alternative can emerge at scale will depend on ecosystem an tool adoption. Nonetheless, it is certainly a worthwile goal. The manuscript itself is concise and well-written. I have several questions and suggestions for the authors.

Questions / suggestions:

> ***One key reference that is missing from the current manuscript is "Scaling read aligners to hundreds of threads on general-purpose processors"[^1], by Langmead et al. It is directly relevant to the current manuscript and describes precisely the scalability bottleneck upstream of multi-core read alignment that arises from the fundamentally unparallelizable nature of parsing a (potentially compressed) FASTQ file.***

Yes, good addition. Added this citation to the introduction.

> ***A question that arises to me in the format is the extent to which assuming that sequence records have either 1 or 2 segments may be problematic. Certain protocols, though relatively rare, make use of more than 2 segments per record to encode information (e.g. some single-cell ATAC-seq protocols have 3 segments per record, with the first 2 representing a biological paried-end read, and the third representing a technical barcode). The authors should consider if and how this might be supported in binseq (presumably vbinseq) or justify excluding support. It seems to me it would be fairly easy to have the number of segments per records be a simple field recorded in the header, and that having values larger than 2 doesn't seem to pose any fundamental tension with the rest of the format (as long as it is fixed throughout the whole file).***

This is an excellent point about multi-segment records. We agree that supporting more than two segments per record would enhance format flexibility, and they are correct that this could be implemented by making the segment count a header field without fundamental tension with the format design. However, we respectfully suggest this falls outside the scope of the initial BINSEQ specification and implementation for several reasons.

First, protocols requiring >2 segments represent a small fraction of current sequencing applications. Second, for the specific example mentioned (single-cell ATAC-seq with technical barcodes), practical workarounds exist within the current specification—the third short segment (<32bp) can be encoded in the 64-bit flag field. Third, and most importantly, properly supporting arbitrary segment counts would require careful consideration of use cases, testing across multiple protocols, and community feedback to ensure the implementation meets real-world needs rather than theoretical flexibility.

We view this as an excellent candidate for a future format extension once we have established the core format in practice and can gather feedback from early adopters about their multi-segment requirements. We have added a note about this potential extension to the Discussion and welcome community input on this feature.

> ***Related to the above, what replacement policy was used in the experiments conducted in the paper?***

For the paper all experiments made use of the random replacement policy to keep the total number of records equal between the different formats.

> *In general, I think a somewhat deeper exploration of the effect of different ambiguous base policies should be conducted, or an alternative ambiguous base policy should be included. Specifically, the current approach can skip records with ambiguous bases, replace those bases with pseudorandom nucleotides, or replace those bases with a fixed nucleotide. These are all reasonable policies. However, I feel it is important to characterize the effect of such a policy on downstream analysis. For example, if an `N` nucleotide appears in the barcode of a single-cell RNA-seq read, then downstream algorithms may handle this differently than if a fixed nucleotide is present (in fact, by replacing the `N` with a fixed nucleotide, a barcode collision may be artificially introduced). Further, in other analyses, tools may have special ways to handle ambiguous bases (assigning them a different cost during alignment). I suspect that in the vast majority of cases, the policies supported by binseq will have no effect on the subsequent analysis, but that may not always be the case (e.g. variant detection from fairly low-coverage data). This is worth further exploration in the manuscript in my opinion. Likewise, perhaps it makes sense to offer a policy where the ambiguous bases are recorded (either as auxiliary data - a list of positions for a record - or in an alternative encoding that allows more than 4 characters). The same comment also applies to the skipping of quality scores in the basic binseq format.*

This is an insightful observation about the downstream effects of ambiguous base handling policies. We agree this is an important consideration and appreciate the specific examples provided (barcode collision introduction, variant calling from low-coverage data).

However, we respectfully contend that a comprehensive evaluation of policy effects across diverse analytical contexts represents a research project of substantial scope, arguably warranting its own dedicated study. The reviewer correctly identifies the core challenge: the impact of ambiguous base handling is highly application-specific and tool-dependent.

A rigorous treatment would require:

1. Systematic evaluation across multiple application domains (variant calling, RNA-seq quantification, single-cell barcode processing, de novo assembly, etc.)
2. Testing with multiple tools within each domain (each with potentially different internal handling of ambiguous bases)
3. Defining appropriate metrics for each application type
4. Using representative datasets with varying properties

Such a study would necessarily be incomplete given the diversity of bioinformatics applications, yet each additional scenario adds substantial experimental and computational burden as well as specialized domain knowledge.

For the current manuscript, we believe the appropriate scope is demonstrating the format's capabilities and performance characteristics, while providing users with flexible policy options (skip, random replacement, fixed replacement, or preservation via four-bit encoding). This allows practitioners to select the approach most appropriate for their specific application and analytical pipeline. We have clarified the available options and their implications in Section 2.1.1.

We note that this question applies equally to existing formats, compressed FASTQ files with ambiguous bases face the same downstream analytical considerations, which are typically addressed at the application level rather than the format level. We have added a brief discussion of this consideration to the Discussion and welcome future work that systematically evaluates these policies across applications.

Another question that occurred to me is if it is the case that in all experiments in the paper, where speeds are compared over different formats, if the experimental samples processed all consist of individual files or file pairs. That is, for large samples, the input is often spread across multiple files (e.g. a large single-cell RNA-seq experiment may have `sample1_lane1_read1_001`, `sample1_lane1_read_2_001`, `sample1_lane1_read1_002`, `sample1_lane1_read2_002`, ..., etc.). In such cases, it is often possible to parse fastq input truly in parallel, at least at the scale of the number of input file streams. While this is certainly (a) not ubiquitous and (b) not taken advantage of by all tools, I think it would be very useful to the reader to know if any of the samples processed in the benchmarks exhibited this quality, and if not, to perhaps include some. This will give a more comprehensive overview of the expected throughput benefits of binseq.

This is a great point about real-world data organization across multiple files, which is indeed common in large-scale experiments, particularly single-cell sequencing.

We chose to benchmark individual files or file pairs for several important methodological reasons. First, file-splitting conventions vary substantially across sequencing platforms, experimental designs, and data management practices—some facilities produce single large files, others split by lane, flowcell, or arbitrary chunk sizes. This heterogeneity makes it difficult to define a representative multi-file benchmark that generalizes across the field. Second, and more fundamentally, multi-file parallelism introduces confounding variables (filesystem I/O patterns, file handle management, process scheduling) that obscure the format-specific performance characteristics we sought to isolate. Our goal was to measure the inherent properties of each format's design rather than system-level parallelism strategies that depend on data organization decisions external to the format itself.

Importantly, we note that within-file and between-file parallelism are complementary rather than competitive. In scenarios where data is split across multiple files, BINSEQ formats would provide the demonstrated speedups for each individual file while also allowing traditional file-level parallel processing. The reviewer is correct that many tools do not currently exploit multi-file parallelism automatically, requiring manual orchestration. This represents a different layer of optimization that could benefit all formats equally and is orthogonal to the format-level improvements we demonstrate here.

We have clarified our experimental design in Section 2.3 and added a brief discussion of the relationship between format-level and file-level parallelism to the Discussion.

Finally, I have a specific design suggestion, which the authors may of course feel free to accept or not. While having an index for random block-level access in vbinseq mirrors the way that e.g. SAM/BAM indices work, I actually feel this is a suboptimal design. These indices tend to be tiny and fast to create, which is why we tend to re-create them on demand. However, it does constitute extra work. Further, such indices are separate files and easy to forget or misplace when transferring data (e.g. one could easily imagine a use grepping for `.vbq` files and forgetting to include all of the index files when creating an archive or copying files between machines). Since the indices are only relevant in the context when the entire file exists on disk (i.e. not in the streaming context), I would recommend the authors consider including the index for vbinseq files as a footer.

Here is the proposal: the vbinseq format can allow an optional footer. This footer comes after all of the record blocks. If this footer is present, the file ends in a magic number (which validates the existence of the footer). The word before the magic number then

stores the size of the index, so that the bytes from (EOF - 8 - size_of_magic_number - index_size) until (EOF - 8 - size_of_magic_number) encode the index itself. This seems as it should work because the index can then be calculated in either a streaming manner as the file is created, which is OK because one won't know the index until all blocks have been compressed, and then appended to the end. Likewise, for a file without the footer, one could easily calculate the index as is currently done and then, rather than write it as a separate file, simply append the footer to the input file. I suspect that this approach may be useful as it keeps the index along with the rest of the data, but does so in a manner that doesn't preclude streaming or parallel writing / appending of the record blocks.

This is an excellent suggestion! We agree that keeping the secondary index file was inelegant and could lead to some awkwardness in handling large collections of files. This proposal has been integrated into VBQ.

Minor points:

On page 4, the statement that the encoding and decoding operations are of sub-linear time complexity don't quite seem right. Certainly, the input must be read, which is itself an O(N) operation for an input of length N. Further, even if one is operating a word at a time and achieving N/(W/sigma) work (for W-bit words, a sigma-bit alphabet encoding and a string of N symbols), this is still linear in the length of the input (i.e. it is still O(N), just with a constant c < 1).

This is a fair point. We've removed this statement.

In section 2.2.2 the authors describe the input and file i/o capabilities of their binseq library and the C and C++ bindings. I took a look at the bindings, and I think it is great that the authors provide these. One question that this brought up to me is whether or not binseq is designed to allow streaming input (I think this would be immensely useful). If so, I'd encourage the authors to provide an additional C / C++ function for opening a binseq file for reading apart from `mmap_open`, one that may be properly suited for reading the file in a streaming manner.

We thank the reviewer for this excellent suggestion and for examining the bindings implementation. The reviewer is correct that streaming support is valuable for many workflows.

Since the initial submission, streaming functionality has been implemented for BQ in both Rust and Python, contributed by members of the open-source community. VBQ streaming support is planned for an upcoming release. For C/C++ bindings, we will add dedicated streaming functions once the Rust streaming implementation has been thoroughly validated in production use, ensuring the API is stable before exposing it through the FFI layer.

We have designed BINSEQ as a community-driven project with a well-engineered Rust core that serves as a stable foundation for language-specific extensions. While our expertise lies primarily in Rust development, we have provided basic C/C++ bindings as a starting point that we hope will enable developers with deep C/C++ expertise to build more idiomatic and performant interfaces for their communities. We welcome contributions from C/C++ developers to enhance these bindings with language-specific optimizations, best practices, and additional features.

We have updated Section 2.2.2 to document the current streaming capabilities.

> ***In section 2.3.3 it is stated that minimap2 specifically notes that it is not designed for the use-case of spliced short-read alignment. However, as of release 2.29 this is no longer true[^2]. To be clear, I don't think the authors need to add minimap2-based short-read, spliced alignment benchmarks to their paper, but rather they should remove or amend this particular justification. Since STAR is, by far, one of the most common short-read RNA-seq spliced read aligners, I think it is fine to having testing for this datatype using only that tool***

We have updated Section 2.3.3 to note that minimap2 now supports short-read spliced alignment as of version 2.29, while clarifying that STAR remains the most widely-used tool for this application.

> ***Finally, this may be out of scope for this paper which defines and justifies (well, in my opinion) the BINSEQ format. However, having a new format and generating a lot of new data to place into this format, seems to me an opportunity to improve the state of data provenance tracking and automated metadata propagation. Might the authors consider if it makes sense to have a structured metadata section in the binseq file that can record relevant information about the source of the data (e.g. sequencing data, machine, etc.)? Perhaps the relevant full set of information is not something that the authors feel comfortable specifying unilaterally, which I think makes sense. However, having the format have built-in support for metadata (even flexible metadata where much of it is optional) could, itself, have***

We thank the reviewer for this thoughtful suggestion and for explicitly noting this may be out of scope. We agree that metadata provenance is an important consideration for modern sequencing formats, and the reviewer is correct that specifying the appropriate metadata schema unilaterally would be premature.

The challenge of metadata standardization in genomics is substantial. Different sequencing platforms, experimental protocols, and institutional practices generate heterogeneous metadata with varying levels of standardization (e.g., instrument identifiers, run parameters, library preparation details, quality metrics). Defining a metadata schema that is both sufficiently flexible to accommodate diverse use cases and sufficiently structured to ensure interoperability would benefit significantly from broader community input and consensus-building, similar to efforts like the GA4GH standards or HTSlib.

The BINSEQ format architecture naturally accommodates future metadata extensions. Both BQ and VBQ include reserved header space, and metadata could be incorporated as an optional section between the header and data records without breaking backward compatibility. We view this as an excellent opportunity for community-driven development once the core format has been adopted and users can provide concrete feedback on their metadata requirements.

We have added a note about potential metadata support to the "Looking forward" section of the Discussion and welcome community input on metadata schema development.
