# Supplementary material for: BINSEQ: A family of high-performance binary formats for nucleotide sequences
Source: PLoS Comput Biol. 2026 May 28;22(5):e1014181. doi: 10.1371/journal.pcbi.1014181 (PMC13232939; doi:10.1371/journal.pcbi.1014181)
Supplement: Attachment — Submitted filename: Reviewer Responses.pdf [file pcbi.1014181.s011.pdf]

We thank the editor and all three reviewers for their thoughtful and constructive feedback. We are pleased that the reviewers find the manuscript substantially improved and appreciate their continued engagement with the work. Below we address each point raised in this minor revision.

Editor Comments

> Reviewer 1 suggests one last additional test on real data to be done, but they explicitly suggest a smaller scope than the other tests/benchmarks in the paper. Reviewer 3 suggests additional discussion of trade-offs related to multi-file inputs and I/O saturation.

We have addressed both points as described below. We have added real-data benchmarks across three representative datasets (Section 2.3.2, Section 3.2, Table 4) and expanded the Discussion with paragraphs addressing multi-file I/O saturation and parallel gzip decompression tools.

Reviewer 1

Point 1: Benchmark on real data in VBQ lossless mode

> Please benchmark on real data in the VBQ lossless mode. They do not need to do comprehensive evaluation like Fig. 2. Just gzip/VBQ file sizes and decompression speed would suffice. Ideally, they could include short genomic reads, long genomic reads and short RNA-seq reads.

We have added a new real-data evaluation (Section 2.3.2, Section 3.2, Table 4) benchmarking VBQ in lossless mode (four-bit encoding with quality scores and sequence headers) across three public datasets of:

1. short-read single-cell RNA seq (SRR11214033; Replogle et al. 2020)
2. short-read WGS (SRR36606595; Illumina NovaSeq X Plus, 2x150bp)
3. long-Read DNA (SRR37455459; PacBio HiFi)

We do a comparison of BQ and lossless VBQ for each of these (excluding BQ for long-read) for single-threaded and multi-threaded parsing.

The results are consistent with our synthetic benchmark, but we agree that this is an excellent addition to the manuscript.

Point 2: Does mmr support separate I/O threads?

> Does mmr support separate I/O threads? If not, it would be good to show how minimap2 works given gzip'd input.

We have added a clarification to the Methods (Section 2.3.4, Alignment) explaining that our mmr implementation does use a dedicated reader thread that feeds records to the alignment worker pool, mirroring the I/O threading strategy of the standard minimap2 command-line tool. For the FASTQ path, mmr uses the paraseq library for parsing in the reader thread, while for BINSEQ inputs, record blocks are dispatched directly to workers for parallel processing. This ensures that the comparison reflects differences in data access efficiency rather than differences in I/O threading strategy.

Point 3: Look beyond your own environment (lossless mode)

> I encourage the authors to look beyond their own environment. In the wider ecosystem, WGS data dominates in terms of data volume and most production teams would choose a lossless format.

We appreciate this perspective and agree that it is important to support the lossless format first. The real-data benchmarks described above (Point 1) specifically evaluate VBQ in lossless mode with quality scores and sequence headers preserved, directly addressing this concern.

Reviewer 2

> The changes made to VBQ to optionally include base qualities, read names (--archive), as well as the possibility to use four-bit encoding for Ns have largely addressed my concerns with the applicability of the BQ and VBQ file formats. I thank the authors for implementing these changes.

We thank the reviewer for their positive assessment and for the suggestions in the previous round that motivated these improvements.

Reviewer 3

Point 1: Multi-file I/O saturation discussion

> While it is true that the improved throughput of Binseq can conceptually be stacked with the improved performance that comes from handling multiple input files in parallel, it is also true that, for sufficiently large numbers of individual input files, I/O may become saturated anyway.

We agree that this is important and understand that our last addition on this point wasn't complete enough. We've added more text to the discussion that addresses this specifically by describing how when there are enough existing file handles to saturate the IO then BINSEQs advantage is narrowed.

Point 2: Mention pugz and rapidgzip

> There exist a couple of tools that support parallel decompression; namely, pugz and rapidgzip. Both of these approaches are based on speculative decompression and/or caching, and therefore trade-off extra computation in order to achieve parallel decompression.

We have also added citations to both of these methods and discuss them as well though we did not add additional benchmarks to include them as they are not currently integrated into standard bioinformatics pipelines or sequence parsing libraries, and thus do not reflect the decompression performance that users encounter in practice.

Additional Changes

Updated synthetic benchmarks: The BINSEQ library has undergone performance optimizations since the previous submission. We have re-run the sequence access benchmarks and updated Tables 2 and 3 and Figure 2 accordingly. Notable improvements include VBQ parallel speedup increasing from 16x to 35x for single-end records and from 16x to 43x for paired-end records.

Paired-end results table: We now include a full paired-end results table (Table 3) in the main text, where BQ achieves 121.71x speedup and VBQ achieves 43.50x speedup over FASTQ.GZ.
